# Adiponectin/leptin ratio as a predictor of acute rejection in early post-transplant period in patients after kidney transplantation

**DOI:** 10.3389/fmed.2023.1117819

**Published:** 2023-02-17

**Authors:** Karol Graňák, Matej Vnučák, Monika Beliančinová, Patrícia Kleinová, Margaréta Pytliaková, Marián Mokáň, Ivana Dedinská

**Affiliations:** ^1^Transplant Centre, University Hospital Martin, Martin, Slovakia; ^2^Department of Internal Medicine, University Hospital in Martin, Jessenius Faculty of Medicine, Comenius University, Martin, Slovakia; ^3^Department of Gastrointestinal Internal Medicine, University Hospital Martin, Jessenius Faculty of Medicine, Comenius University, Martin, Slovakia

**Keywords:** adiponectin/leptin ratio, kidney transplantation, acute rejection, antibody-mediated rejection, adipose tissue hormones

## Abstract

**Introduction:**

Adipokines are largely involved in the regulation of immune system activity. While leptin is the main pro-inflammatory marker of adipose tissue, adiponectin is characterized by anti-inflammatory effects. The aim of our study was to determine the risk of acute graft rejection in protocol biopsy depending on the adiponectin/leptin (A/L) ratio in patients after kidney transplantation (KT).

**Materials and methods:**

A total of 104 patients were included in the prospective analysis, in whom the levels of adipokines were examined pre-transplant, in the 3rd month after KT and the A/L ratio was calculated. In the 3rd month after KT, all patients underwent protocol biopsy of the graft and examination of donor-specific antibodies (DSA) using the Luminex method.

**Results:**

After adjusting for differences in the basic characteristics of the donor and recipient, we identified a subgroup with A/L ratio < 0.5 pre-transplant [HR 1.6126, (*P* = 0.0133)] and 3 months after KT [HR 1.3150, (*P* = 0.0172)] as independent risk factor for acute graft rejection. In the subsequent specification of the rejection episode, we identified the risk ratio A/L < 0.5 before KT [HR 2.2353, (*P* = 0.0357)] and 3 months after KT [HR 3.0954, (*P* = 0.0237)] as independent risk factor for the development of acute humoral rejection with DSA positivity.

**Conclusion:**

This is the first study to investigate the relationship between A/L ratio and immunological risk in terms of the development of rejection changes in patients after KT. In our study, we found that A/L ratio < 0.5 is an independent risk factor for the development of acute humoral rejection and *de novo* DSA production in the third month after KT.

## Introduction

It is well known today that adipose tissue is involved in the production and secretion of a wide range of bioactive peptides, known as adipokines, which have a local (paracrine) but also a systemic (endocrine) effect. In addition to these efferent signals, adipose tissue receives signals from hormonal systems or the central nervous system through many receptors. Thanks to this interactive network, adipose tissue is directly involved in the coordination of biological processes, including energy metabolism and immune functions (1). Most molecules, especially those secreted by the non-adipocyte fraction of adipose tissue, have a dominant paracrine effect. Leptin and adiponectin are today generally accepted as the only endocrine hormones of adipose tissue with a defined effect on target organs (2). Their presence in the human body correlates with the amount of adipose tissue. Adipokines can be classified in different ways, but from the point of view of their impact on the immune system, we divide them into two groups - pro-inflammatory and anti-inflammatory (3).

Leptin is considered a major pro-inflammatory marker and shares structural homology with the interleukin (IL)-6 receptor. It participates in the activation and proliferation of granulocytes, monocytes, macrophages, dendritic cells, natural killer cells and leads to increased production of pro-inflammatory cytokines (IL-6, IL-12, tumor necrosis factor—TNF) (4). Leptin is directly involved in the regulation of activation and differentiation of T and B cells. It supports the proliferation of naïve and memory T cells and increases the secretion of Th1 and Th17 lymphocytes. Mechanism studies have shown that leptin activates the mTOR pathway, thereby having a positive effect on CD4 + CD25 + FOXP3 + effector T cells. In addition, it stimulates the formation, maturation and survival of thymic T cells by reducing their apoptosis (5). Its important role has also been demonstrated in the regulation of the development and function of B cells. Since the receptor for leptin is expressed on B cells, its direct effect is assumed. Deficiency of leptin signaling led to a reduction of B cells in the bone marrow and peripheral blood, and its reduced level was associated with a lower representation of pro-B and immature B cells in the bone marrow (6).

Adiponectin, as the main representative of the group of anti-inflammatory markers of adipose tissue, acts through two receptors, AdipoR1 and AdipoR2, which, among many other tissues, are also found on most cells of the immune system (7). Functionally, adiponectin reduces the ability of macrophages to phagocytose and secrete pro-inflammatory cytokines, while increasing the production of anti-inflammatory IL-10 (3). In endothelial cells, it blocks the expression of adhesive molecules, which results in a reduction of the diapedesis of circulating monocytes (4). Several studies have shown that adiponectin is a negative regulator of T cell activity. It has been shown to inhibit proliferation and cytokine production of T cells and promote their apoptosis. Recent data also suggest that it is involved in the inhibition of Th1 and Th17 lymphocyte differentiation (8). Even though the immunomodulatory effect of adiponectin on B lymphocytes is not completely clear, it has been shown to inhibit B lymphopoiesis in long-term bone marrow cultures. In addition, adiponectin stimulates B cells to secrete the peptide PEPITEM, which specifically inhibits the migration of CD4+ and CD8+ memory T cells (9).

Recent studies suggest that patients with adipose tissue dysfunction, characterized by lower adiponectin secretion compared to leptin levels, have an increased cardiometabolic risk. This fact results from an increase in systemic inflammation and oxidative stress, the occurrence of which is negatively correlated with the A/L ratio (10). A/L ratio can be a practical marker characterizing adipose tissue dysfunction. From the results on the general population, it follows that an A/L ratio >1 can be considered normal, an A/L ratio of 0.5–1 indicates a moderate risk and an A/L ratio <0.5 a high risk (11). Due to the significant involvement of adipokines in the regulation of immune system processes, we assume that the A/L ratio could be correlated with the occurrence of immune-related reactions in the transplanted population. If confirmed, it would be possible to identify recipients at increased risk of developing acute graft rejection. The aim of our study was to determine the risk of acute graft rejection in protocol biopsy depending on the A/L ratio in patients after KT.

## Materials and methods

Adult patients who underwent primary KT at the Martin Transplantation Center in 2018–2019 were included in our prospective monocenter study. Patients with a history of diabetes mellitus (DM) type I or II, those who died during the study period, patients who suffered from infectious complications and those who did not undergo a protocol graft biopsy (poor anatomical conditions, recurrent infections) were excluded from the follow-up. A total of 104 patients completed our prospective follow-up.

All patients in the studied sample were set on the same immunosuppressive protocol. As part of the induction protocol, they were administered antithymocyte globulin in a cumulative dose of 3.5 mg/kg of body weight, which was divided into three doses (pre-transplantation, day 1 and day 2 after KT). A triple combination was used in the prophylactic immunosuppressive regimen: tacrolimus, mycophenolic acid and corticosteroids. Methylprednisolone was applied at a dose of 500 mg intravenously pretransplantation and on the first posttransplantation day followed by a change to oral prednisone (prednisone 20 mg until the second week after KT, prednisone 15 mg until the fourth week after KT, prednisone 10 mg until 16 weeks after KT and prednisone 7.5 mg up to 12 months after KT). Mycophenolic acid was used in a total daily dose of 1,440 mg until the first month after KT, in a daily dose of 1,080 mg until the third month after KT, and then continued with a daily dose of 720 mg.

We examined the basic level of leptin, adiponectin, IL-6, and IL-10 in KT recipients at the time of flow cytometry crossmatch (FXCM), i.e., approximately 4–5 h before the transplantation. In the post-transplantation period, we examined their levels at 3 months, i.e., at the time of the protocol biopsy of the graft. We used Human Leptin Quantikine ELISA Kit, Human Total Adiponectin ELISA Kit, LEGEND MAX Human IL-6 ELISA Kit and LEGEND MAX Human IL-10 Kit to investigate the levels of adipokines and interleukins. The A/L ratio was calculated from the measured values. We consider an A/L ratio above 1.0 to be normal, an A/L ratio of 0.5–1.0 indicates a moderate risk, and an A/L ratio <0.5 a high metabolic risk (12).

At the time of KT, we recorded: basic characteristics of the donor (donor with extended criteria, cold ischemia time) and characteristics of the recipient (age, sex, length of dialysis treatment, underlying cause of kidney failure, delayed onset of graft function, panel of reactive antibodies, number of mismatches in class A, B, DR, and DQ). At 3 months after KT, we also determined anthropometric parameters (waist circumference, body mass index—BMI), glucose metabolism parameters (c-peptide and immunoreactive insulin levels), lipid profile (total cholesterol, low-density lipoprotein—LDL, high-density lipoprotein—HDL, triglycerides), vitamin D, tacrolimus level and parameters reflecting graft function as glomerular filtration rate determined using the CKD-EPI (Chronic Kidney Disease—Epidemiology Collaboration Index) formula and quantitative proteinuria from 24-h urine collection.

Protocol biopsy of the graft and examination of DSA was performed during a short hospitalization at 3 months after KT in all patients included in our study. Biopsy was performed under ultrasonographic control using an 18-gauge puncture needle. All samples were histologically evaluated by the same pathologist. We then divided the studied sample according to the result of the histological examination (based on the Banff classification from 2019) into a group with a negative result, with findings of interstitial fibrosis and tubular atrophy (IFTA), acute tubular necrosis (ATN), acute cellular rejection (ACR) including borderline changes and antibody-mediated rejection (AMR) with DSA positivity. The examination of DSA was carried out using the LUMINEX methodology, when a value of ≥500 mean fluorescence intensity (MFI) was considered a positive result.

In our study, we used a certified statistical program, MedCalc version 13.1.2. (MedCalc Software VAT registration number BE 0809 344,640, Member of International Association of Statistical Computing, Ostend, Belgium). Using parametric (Student’s *t*-test) or non-parametric tests we compared continuous variables between groups; the χ^2^ test and Fisher’s exact test were used to analyze associations between categorical variables, as appropriate. For non-parametric tests, we used the Wilcoxon test in the first step (Table 1) to compare the group of patients before KT and 3 months after KT. In subsequent analysis (Tables 2, 3), we used the Mann–Whitney test to compare independent groups according to A/L ratio. To perform multivariate analysis, we used Cox regression Hazard model. A *P*-value <0.05 was considered to be statistically significant.

**TABLE 1:** Basic study file characteristics.

*n* = 104	Base line	3M	*P*-value
Men (%)	63.5 (*n* = 66)	–	–
Age at the time of KT (Y)	45 ± 11	–	–
BMI (kg/m^2^)	26 ± 4	25.8 ± 4.2	0.7255
Waist circumference (cm)	–	94 ± 12.4	–
ECD (%)	25 (*n* = 26)	–	–
CIT (min)	745 ± 388	–	–
DGF (%)	10.5 (*n* = 11)	–	–
Time of dialysis treatment (M)	27 (median 15)	–	–
PRA (%)	1.6 ± 0.7	–	–
Mismatch A	1.3 ± 0.7	–	–
Mismatch B	1.4 ± 0.6	–	–
Mismatch DR	1.3 ± 0.6	–	–
Mismatch DQ	1.1 ± 0.8	–	–
Leptin (ng/ml)	44.8 ± 27.8	53.6 ± 29.2	0.0271
Adiponectin (μ g/ml)	18.8 ± 8.8	15.9 ± 9	0.0197
Adiponectin/leptin ratio	0.41 ± 0.3	0.3 ± 0.3	0.0088
IL-6 (pg/ml)	26.3 ± 11.4	34.2 ± 18	0.0002
IL-10 (pg/ml)	5.6 ± 3.2	7.2 ± 4	0.0017
Glycemia (mmol/L)	–	5.7 ± 1.6	–
PTDM (%)	–	23.1 (*n* = 24)	–
IFG (%)	–	7.7 (*n* = 8)	–
IGT (%)	–	30.8 (*n* = 32)	–
HbA1c (%)	–	3.7 ± 0.9	–
C–peptid (μ g/L)	–	4.2 ± 2	–
IRI (mU/L)	–	8.1 ± 3.6	–
Cholesterol (mmol/L)	–	5.4 ± 1.3	–
LDL (mmol/L)	–	3.1 ± 1.1	–
HDL (mmol/L)	–	1.4 ± 0.5	–
Triglycerides (mmol/L)	–	2.5 ± 1.5	–
TAC value (ng/L)	–	8.8 ± 3.2	–
eGFR CKD-EPI (ml/min)	–	55.2 ± 21.7	–

KT, kidney transplantation; BMI, body mass index; ECD, expanded criteria donor; CIT, cold ischemia time; DGF, delayed graft function; PRA, panel reactive antibodies; IL, interleukin; PTDM, posttransplant diabetes mellitus; IFG, impaired fasting glucose; IGT, impaired glucose tolerance; HbA1c, glycated hemoglobin; IRI, immunoreactive insulin; LDL, low density lipoprotein; HDL, high density lipoprotein; TAC, tacrolimus; eGFR CKD EPI, estimated glomerular filtration rate by Chronic Kidney Disease Epidemiology Collaboration Index; Y, year; M, month.

**TABLE 2:** Comparison of the observed groups based on A/L ratio baseline.

*n* = 104	A/L ratio < 0.5 *n* = 45	A/L ratio 0.5-1 *n* = 21	A/L ratio > 1 *n* = 38	*P*-value*	*P*-value**	*P*-value***
Men (%)	57.8	38.1	84.2	0.1388	0.0003	0.0094
Age at the time of KT (Y)	45.7 ± 13.4	44.6 ± 8.8	44.7 ± 10.8	0.7331	0.9712	0.7126
BMI (kg/m^2^)	27.9 ± 3.9	25.1 ± 3.7	25 ± 4.4	0.0075	0.9300	0.0021
ECD (%)	33.3	19	18.4	0.2354	0.9552	0.1276
CIT (min)	928 ± 380	757 ± 364	550 ± 420	0.0893	0.0629	<0.0001
DGF (%)	13.3	9.5	7.9	0.6616	0.8340	0.4330
Time of dialysis treatment (M)	31.5 ± 17	30.2 ± 14	19.3 ± 13.1	0.7613	0.0042	0.0005
PRA (%)	2 ± 1	1.5 ± 0.7	1.3 ± 0.4	0.0431	0.4866	0.0001
Mismatch	1.3 ± 0.7	1.5 ± 0.4	1.2 ± 0.7	0.2282	0.0766	0.5185
IL-6 base line (pg/ml)	31.7 ± 13	22.8 ± 8.1	24.4 ± 13.1	0.0054	0.6137	0.0139
IL-10 base line (pg/ml)	4.3 ± 3.9	5.8 ± 2.5	6.7 ± 3.2	0.1121	0.2703	0.0033

KT, kidney transplantation; BMI, body mass index; ECD, expanded criteria donor; CIT, cold ischemia time; DGF, delayed graft function; PRA, panel reactive antibodies; IL, interleukin; A/L, adiponectin/leptin; Y, year; M, month.

*A/L ratio < 0.5 vs. A/L ratio 0.5-1.

**A/L ratio 0.5-1 vs. A/L ratio > 1.

***A/L ratio < 0.5 vs. A/L ratio > 1.

**TABLE 3:** Comparison of the observed groups based on A/L ratio at 3 months after kidney transplantation.

*n* = 104	A/L ratio < 0.5 *n* = 49	A/L ratio 0.5-1 *n* = 17	A/L ratio > 1 *n* = 38	*P*-value*	*P*-value**	*P*-value***
Men (%)	57.1	35.3	84.2	0.1241	0.0006	0.0003
Age at the time of KT (Y)	46.2 ± 13.8	43.8 ± 9.6	45 ± 9.6	0.5103	0.6701	0.6489
BMI 3M (kg/m^2^)	28.6 ± 3.7	24.3 ± 5.8	23.7 ± 2.8	0.0008	0.6051	<0.0001
Waist circumference 3M (cm)	101.3 ± 10.9	89.3 ± 16.8	91.4 ± 9.8	0.0013	0.5622	<0.0001
ECD (%)	34.7	11.8	18.4	0.0746	0.4959	0.5446
CIT (min)	874 ± 382	694 ± 352	667 ± 430	0.0945	0.8215	0.0199
DGF (%)	14.3	11.8	5.3	0.7974	0.4475	0.3964
Time of dialysis treatment (M)	30.1 ± 16.1	30.2 ± 14	20.7 ± 14	0.9819	0.0239	0.0054
PRA (%)	2 ± 1	1.5 ± 0.7	1.3 ± 0.4	0.0617	0.1843	0.0001
Mismatch	1.3 ± 0.6	1.5 ± 0.4	1.2 ± 0.7	0.2065	0.1058	0.4755
IL-6 3M (pg/ml)	42.6 ± 24	33 ± 14	27 ± 16	0.1249	0.1882	0.0009
IL-10 3M (pg/ml)	6.2 ± 3.8	7.5 ± 4.4	7.5 ± 3.8	0.2477	1.0000	0.1172
TAC value (ng/ml)	8.5 ± 3.6	9.2 ± 4	8.7 ± 2	0.5044	0.5375	0.7593
eGFR CKD-EPI 3M (ml/min)	51.3 ± 21.7	50.1 ± 22.9	64.2 ± 20.5	0.8470	0.0271	0.0060
ACR 3M (%)	12.2	23.5	7.9	0.2660	0.1649	0.1119
AMR 3M (%)	14.3	17.6	0	0.7456	0.0156	0.0084

KT, kidney transplantation; BMI, body mass index; ECD, expanded criteria donor; CIT, cold ischemia time; DGF, delayed graft function; PRA, panel reactive antibodies; IL, interleukin; TAC, tacrolimus; eGFR CKD-EPI, estimated glomerular filtration rate by Chronic Kidney Disease – Epidemiology Collaboration Index; ACR, acute cellular rejection; AMR, antibody mediated rejection; A/L, adiponectin/leptin, Y, year; M, month.

*A/L ratio < 0.5 vs. A/L ratio 0.5-1.

**A/L ratio 0.5-1 vs. A/L ratio > 1.

***A/L ratio < 0.5 vs. A/L ratio > 1.

## Results

A total of 170 patients after primary deceased donor KT were primarily included in the study. 66 patients were excluded from the follow-up meeting the exclusion criteria that we stated in the materials and methods section. Thus, 104 patients completed our prospective follow-up.

The serum level of tacrolimus was maintained during the monitored period in the range of 10–15 ng/L during the first month after KT, then in the range of 8.0–10 ng/L until the third month after KT. We did not observe significant differences in the level of tacrolimus between the individual studied subgroups. Likewise, there was no significant difference in the daily dose of prednisone.

Table 1 summarizes the basic characteristics of the investigated file. Of the total number of patients, 63.5% were men and the average age was 45 ± 11 years. When comparing the average serum levels of adipokines and interleukins at the beginning and in the third month of follow-up, we found that in the third month after KT there was a significant increase in inflammatory markers (leptin, IL-6, IL-10) and, conversely, a significant decrease in anti-inflammatory marker (adiponectin). The A/L ratio decreased significantly during this period (Table 1).

We primarily divided the studied group into three subgroups based on the A/L ratio before transplantation and in the third month after KT: 1. A/L ratio <0.5, 2. A/L ratio 0.5–1.0, 3. A/L ratio >1.0. We compared the subgroups among themselves according to parameters related to the eventual development of graft rejection. Table 2 shows the comparison before KT. We found that there were significantly more men in the subgroup with A/L ratio > 1, on the other hand, there was no difference in the age structure between the individual subgroups. In the subgroup with high metabolic risk (A/L < 0.5), we found a significantly higher BMI value compared to subgroup with A/L ratio > 1, but also with A/L ratio 0.5–1.0. In the mentioned subgroup, patients also had a significantly higher panel reactive antibodies (PRA) value. In the subgroup with a normal A/L ratio, patients showed significantly shorter cold ischemia time and shorter time spent in the dialysis program. The level of IL-6 was significantly higher in the high-risk subgroup (A/L < 0.5), and the level of IL-10, on the other hand, was significantly lower in this subgroup compared to the subgroup with A/L ratio > 1.0. We did not notice a difference in the occurrence of delayed onset of graft function (DGF) or in the representation of donors with extended criteria (Table 2).

Table 3 summarizes a comparison of these subgroups in the third month after KT. As before transplantation, there were significantly more men in the subgroup with A/L ratio > 1.0 in the third month after KT and the age structure also did not change. Patients with an A/L ratio < 0.5 had a significantly higher BMI value, but also a waist circumference value compared to other subgroups. In this high-risk subgroup, patients had a higher PRA value. Patients in the subgroup with A/L ratio > 1.0 spent a significantly shorter time in the hemodialysis program and, compared to the subgroup with A/L ratio < 0.5, had a significantly shorter time of cold ischemia. The level of IL-6 was also significantly higher in the subgroup with A/L ratio < 0.5, on the other hand, the level of IL-10 did not show any significant differences between the subgroups. Using the eGFR value, a significantly better graft function was detected at 3 months after KT in the subgroup with a normal A/L ratio compared to the other subgroups. At the same time, we identified a significantly lower incidence of AMR in this subgroup (A/L > 1.0) compared to the high-risk (A/L < 0.5) and medium-risk (A/L ratio 0.5 – 1.0) subgroups. There were no differences in the incidence of ACR (Table 3).

In the multivariate analysis, we used the Cox regression hazard model. After adjusting for differences in the basic characteristics of the donor and recipient, we identified the length of dialysis treatment more than 24 months as an independent risk factor for A/L ratio < 0.5 pre-transplant [HR 2.2727, (*P* = 0.0386)] and BMI value > 30 kg/m^2^ as independent risk factor for A/L ratio < 0.5 in the third month after KT [HR 3.8235, (*P* = 0.0386)] (Tables 4, 5).

**TABLE 4:** Cox proportional hazard model, end point: A/L ratio < 0.5 baseline.

A/L < 0.5 base line	HR	95% CI	*P*
Men	1.6825	0.5708–4.9599	0.3455
Age at the time of KT ≥ 50 Y	4.7406	2.1386–10.5059	0.9647
BMI base line ≥ 30 kg/m^2^	1.5001	0.2744–8.1897	0.6396
Time of dialysis treatment > 24 M	2.2727	1.0439–4.9481	0.0386
PRA > 1%	1.0645	0.3675–3.0838	0.9083
hyper IL-6 base line	2.3703	1.0693–4.6525	0.9666
hypo IL-10 base line	0.8902	0.1825–4.3414	0.8859

A/L, adiponectin/leptin; Y, year; M, month; PRA, panel reactive antibodies; IL, interleukin. Hyper IL-6: IL-6 value > 7.5 pg/ml; Hypo IL-10: IL-10 value < 10 pg/ml; Age limit: based on the average age of the group; Time of dialysis treatment limit: based on the average time of the group; PRA limit: based on the average PRA value of the group.

**TABLE 5:** Cox proportional hazard model, end point: A/L ratio < 0.5 at 3 months after kidney transplantation.

A/L < 0.5 M3	HR	95% CI	*P*
Men	0.9545	0.3243–2.8096	0.9326
Age at the time of KT ≥ 50 Y	1.5986	0.5474–4.6680	0.3909
BMI 3M ≥ 30 kg/m^2^	3.8235	1.5255–9.5834	0.0042
Waist circumference (men ≥ 94 cm, women ≥ 80 cm)	1.4519	0.2174–9.6962	0.1481
ECD	0.9976	0.1399–7.1119	0.9981
CIT ≥ 12 hod	1.3577	0.4909–3.7548	0.5558
DGF	1.2552	0.2569–6.1325	0.7788
Time of dialysis treatment > 24 M	1.3090	0.4757–3.6020	0.6021
PRA > 1%	0.8728	0.2765–2.7552	0.8165
hyper IL- 6 M3	2.7447	0.3165–23.8046	0.3596
hypo IL-10 M3	0.8961	0.2422–3.3150	0.8694
eGFR CKD-EPI M3 < 60 ml/min	2.5623	0.4236–15.4993	0.3056

A/L, adiponectin/leptin; Y, year; M, month; PRA, panel reactive antibodies; IL, interleukin; ECD, expanded criteria donor; CIT, cold ischemia time; DGF, delayed graft function; eGFR CKD-EPI, estimated glomerular filtration rate by Chronic Kidney Disease – Epidemiology Collaboration Index.

Hyper IL-6: IL-6 value > 7.5 pg/ml; Hypo IL-10: IL-10 value < 10 pg/ml; Age limit: based on the average age of the group; Time of dialysis treatment limit: based on the average time of the group; PRA limit: based on the average PRA value of the group; CIT limit: based on the average CIT value of the group; eGFR limit: based on the average eGFR value of the group; Waist circumference limit: based on the IDF criteria (men ≥ 94 cm, women ≥ 80 cm).

In the next step, we used the Cox regression Hazard model to determine independent risk factors for the occurrence of acute rejection in protocol graft biopsy. After adjusting for differences in the baseline characteristics of the donor and recipient, we identified a subgroup with an A/L ratio < 0.5 pretransplant [HR 1.6126, (*P* = 0.0133)] and 3 months after KT [HR 1.3150, (*P* = 0.0172)] as an independent risk factor for the occurrence of acute graft rejection (ACR + AMR) (Table 6). In the subsequent specification of the rejection episode, we identified the risk ratio A/L < 0.5 before transplantation [HR 2.2353, (*P* = 0.0357)] and 3 months after KT [HR 3.0954, (*P* = 0.0237)] as an independent risk factor for the development of AMR with DSA positivity (Table 7). On the contrary, the investigated A/L ratios were not detected as independent risk or protective factors for the development of ACR (Table 8).

**TABLE 6:** Cox proportional hazard model, end point: acute rejection in protocol graft biopsy.

Rejection (ACR + AMR)	HR	95% CI	*P*
Hyper IL-6 base line	0.1795	0.0187–1.7247	0.1368
Hypo IL-10 base line	1.6744	0.2095–13.3851	0.6269
Adiponectin/leptin ratio base line < 0.5	1.6126	1.3530–1.9798	0.0133
Adiponectin/leptin ratio base line 0.5–1	1.3893	0.3730–2.5743	0.6240
Adiponectin/leptin ratio base line > 1	1.1250	0.5455–2.3200	0.7499
Hyper IL-6 3M	0.2317	0.0224–2.3953	0.2198
Hypo IL-10 3M	1.7000	0.4252–6.7972	0.4530
Adiponectin/leptin ratio 3M < 0.5	1.3150	1.0169–1.7094	0.0172
Adiponectin/leptin ratio 3M > 1	1.3969	0.3751–5.2016	0.6183

ACR, acute cellular rejection; AMR, antibody-mediated rejection; IL, interleukin; M, month.

Hyper IL-6: IL-6 value > 7.5 pg/ml; Hypo IL-10: IL-10 value < 10 pg/ml.

**TABLE 7:** Cox proportional hazard model, end point: antibody-mediated rejection in protocol graft biopsy.

AMR	HR	95% CI	*P*
Hyper IL-6 base line	0.5386	0.0337–8.6109	0.6617
Hypo IL-10 base line	6.7241	4.8469–8.3160	0.9510
Adiponectin/Leptin ratio base line < 0.5	2.2353	1.4094–3.2031	0.0357
Adiponectin/Leptin ratio base line 0.5–1	0.8710	0.1593–4.7404	0.8689
Adiponectin/Leptin ratio base line > 1	0.9027	0.7939–1.6399	0.8570
Hyper IL-6 3M	0.4617	0.0289–7.3812	0.5847
Hypo IL-10 3M	0.2160	0.0252–1.8489	0.1618
Adiponectin/Leptin ratio 3M < 0.5	3.0954	1.9346–6.8478	0.0237
Adiponectin/Leptin ratio 3M 0.5–1	0.2724	0.0672–1.9113	0.9584

AMR, antibody-mediated rejection; IL, interleukin; M, month.

Hyper IL-6: IL-6 value > 7.5 pg/ml; Hypo IL-10: IL-10 value < 10 pg/ml.

**TABLE 8:** Cox proportional hazard model, end point: acute cellular rejection in protocol graft biopsy.

ACR	HR	95% CI	*P*
Hyper IL-6 base line	0.7547	0.6034–0.9703	0.9639
Hypo IL-10 base line	0.6280	0.0653–6.0421	0.6871
Adiponectin/Leptin ratio base line < 0.5	1.1172	0.1574–7.9314	0.9118
Adiponectin/Leptin ratio base line 0.5–1	1.7365	0.2446–2.3281	0.5810
Adiponectin/Leptin ratio base line > 1	0.9649	0.1169–2.5643	0.0863
Hyper IL-6 3M	7.167	8.3882–10.8280	0.9630
Hypo IL-10 3M	5.2431	3.1036–9.3148	0.9255
Adiponectin/Leptin ratio 3M < 0.5	0.8505	0.1198–6.0380	0.8714
Adiponectin/Leptin ratio 3M > 1	0.9089	0.3180–1.9207	0.9660

ACR, acute cellular rejection; IL, interleukin; M, month.

Hyper IL-6: IL-6 value > 7.5 pg/ml; Hypo IL-10: IL-10 value < 10 pg/ml.

**FIGURE 1:**
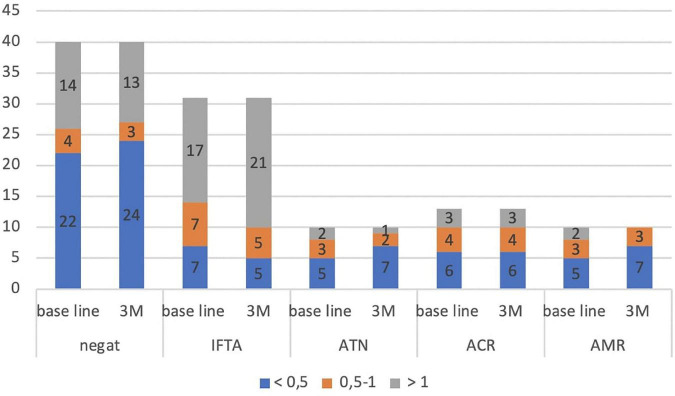
Development of the A/L ratio during 3 months of follow-up in all subgroups (according to histological findings in protocol graft biopsy). IFTA, interstitial fibrosis and tubular atrophy; ATN, acute tubular necrosis; ACR, acute cellular rejection; AMR, antibody-mediated rejection; M, month.

We evaluated the development of the A/L ratio (according to defined subgroups) during the three months of follow-up in all groups according to the histological findings in the protocol biopsy of the graft. In the individual groups, we did not find significant change in the A/L ratio before transplantation and 3 months after KT (Figure 1).

**FIGURE 2:**
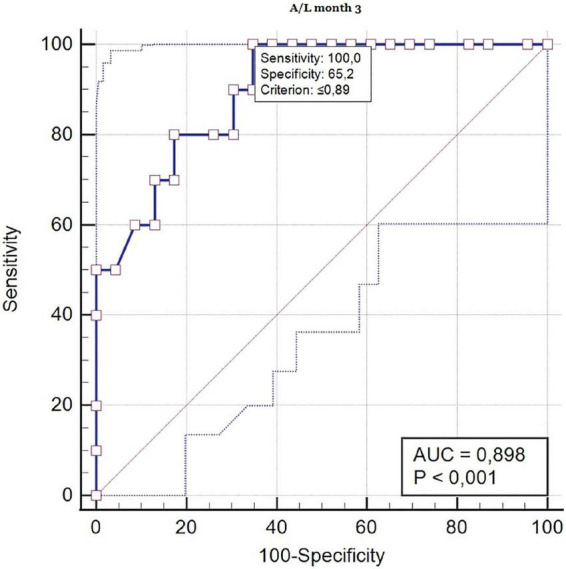
ROC curve analysis (A/L ratio in month 3 as a predictor for AMR 3 months after kidney transplantation). ROC, receiver operating characteristic; AUC, area under the curve.

Finally, we performed the ROC curve analysis for A/L ratio month 3 as a predictor for AMR 3 months after KT with sensitivity 100, specificity 65.2 and criterion ≤ 0.89 (Figure 2).

## Discussion

To our knowledge, this is the first study that investigated the A/L ratio in patients after KT in the context of the risk of developing acute graft rejection. In our work, we found that the A/L ratio < 0.5 pre-transplantation and 3 months after KT represents an independent risk factor for the finding of acute graft rejection in protocol biopsy. At the same time, we specified that the risk ratio A/L < 0.5 was significantly correlated with the development of AMR in protocol biopsy with *de novo* DSA production. This finding was clearly supported by the result of the ROC curve analysis with AUR 0.898, which confirmed significantly larger probability of developing AMR in the group with high-risk A/L ratio.

The A/L ratio can be considered as an indicator of adipose tissue dysfunction and the balance between these adipokines may very likely play an important role in the clinical outcome in this group of patients. Monitoring of adipose tissue hormones in the transplant population has only a short history, and until now they have been investigated as separate variables in correlation with cardiometabolic risk factors. In our recently published work, we found that a high-risk A/L ratio (<0.5) was significantly associated with the occurrence of post-transplant diabetes mellitus (PTDM) and pre-diabetic conditions 1 year after KT (13).

As a pro-inflammatory marker, leptin largely alters the adaptive immune system by activating CD4+ T lymphocytes and negative signaling for CD25+ T regulatory cells (14). Joffre et al. in their work, they assume that the inactivity of regulatory T cells can lead to graft loss, as stimulated CD4 + CD25 + Foxp3 regulatory T cells prevent rejection of the transplanted organ (12). However, this claim has not yet been confirmed by clinical studies. In previous years, worse graft survival was detected in obese patients with hyperleptinemia. Authors Moraes-Vieira et al. they searched for a possible immunological basis in mouse models. An interesting finding was that obese mice that were leptin-deficient showed better survival of skin grafts, which indicated that the transplant outcome observed in obese patients may not be directly related to obesity but to hyperleptinemia. The authors therefore focused on the immunological background and found that CD4+ T cells differentiated more efficiently into T regulatory lymphocytes and showed a lower degree of proliferation *in vivo*, which was ultimately highly likely the cause of better graft survival in these mice (15). No association between leptin and systemic inflammation in patients after KT has been found in observational studies conducted so far (16). On the contrary, in a study on the general population, the conclusions of which were published in 2017 by Fruhbeck et al. a strong negative correlation was found between c-reactive protein level, serum amyloid A and A/L ratio. These findings may indicate that the A/L ratio reflects adipose tissue dysfunction-induced systemic inflammation (10). Work on the anti-inflammatory adiponectin suggests that it is involved in the activation of nuclear factor κB (NF-κB) transcription. NF-κB is a protein kinase that regulates the immune system through the activity of T cells and plays an important role in acute rejection of a vascularized organ or in the etiopathogenesis of several autoimmune diseases. The authors of Vu et al. evaluated the association between NF-κB gene polymorphisms and the outcome of the transplant itself in a sample of 607 Hispanics after KT. Recipients with the NF-κB1 polymorphism had significantly fewer biopsy-verified acute graft rejections (17). Alam et al. in 2013 investigated graft survival in 987 patients after KT based on the adiponectin level. However, an increased level of the protective acting adiponectin was not associated with better graft survival (18).

Fonseca et al. in 2015 were the first to investigate the clinical significance of adipokines in the context of graft dysfunction in patients after KT and not in the context of cardiometabolic complications. In a sample of 40 adult patients who underwent KT, the relationship of leptin, adiponectin levels with DGF and acute rejection were evaluated. Serum levels of adipokines were measured before transplantation and subsequently in the first 7 days after KT. Leptin level was significantly higher in the group of patients who developed DGF compared to those who had prompt onset of graft function. Even serum leptin on the first day after KT predicted DGF slightly better than serum creatinine. Conversely, adiponectinemia was not significantly higher in graft dysfunction and was not a predictor of DGF. In the mentioned study, the authors also monitored the possible prediction of the development of acute graft rejection and the formation of anti-human leukocyte antigen (HLA) antibodies based on leptinemia, but no significant predictive value was found. A possible reason was also the minimal number of patients with acute rejection in the study (19). The importance of adiponectinemia in predicting the development of graft function after KT was presented in the study published by Roos et al. In 206 patients, they examined the level of total adiponectin and the high molecular weight multimer, as its main active form, before transplantation. At a 36-month follow-up, both forms were significantly associated with markers of endothelial dysfunction, arteriosclerosis, and at the same time significantly predicted graft survival. This inverse association between adiponectin and graft survival may be explained, at least in part, by its protective effects on endothelial cells and vascular inflammation (20).

A secondary finding in our study was that recipients who showed a pre-transplant high-risk A/L ratio < 0.5 spent a significantly longer time in a chronic hemodialysis program. The cause is probably chronic inflammation with oxidative stress, which are one of the basic determinants of cardiovascular morbidity and mortality in long-term dialysis patients (21). An unsurprising finding was a significantly higher representation of obese patients (BMI > 30 kg/m^2^) in the group with A/L ratio < 0.5 three months after KT.

Based on our findings, we assume the importance of the level of adipokines in transplant patients, not only for the occurrence of already known cardiometabolic complications, but especially in the context of the development of rejection changes and DSA production. The results of our study indicate that the evaluation of the ratio of these hormones with the opposite effect has a greater clinical significance than monitoring them separately. In clinical practice, the monitoring of the A/L ratio can be an important early predictor of risk groups of recipients for the development of rejection changes, DSA production and, probably, resulting worse function or survival of the graft. However, these claims will require further studies with longer follow-up and a larger sample of patients.

The limitation of this study is the low number of patients included in the individual monitored subgroups. On the other hand, this is the first study that deals with this issue and therefore we consider the sample size for this pilot project to be acceptable. Another limitation may be the absence of previous works on a transplanted sample of patients, so we designed our study based on the findings in the general population cohorts.

## Conclusion

This is the first study to investigate the relationship between A/L ratio and immunological risk in terms of the development of rejection changes in patients after KT. In our study, we found that A/L ratio < 0.5 is an independent risk factor for the development of AMR and *de novo* DSA production in the third month after KT. Based on our findings, we attribute an importance to adipokines not only in the occurrence of metabolic, but especially immunological complications with a possible impact on the survival of grafts. A/L ratio can be an important early indicator of risk groups of patients undergoing KT. Further studies in a larger sample of patients will be needed to confirm our findings.

## Data availability statement

The original contributions presented in this study are included in the article/supplementary material, further inquiries can be directed to the corresponding author.

## Ethics statement

Informed consent for included participants was checked and approved by University Hospital’s and Jessenius Faculty of Medicine’s in Martin, Slovakia, Ethical Committees (EK 33/2018). The patients/participants provided their written informed consent to participate in this study.

## Author contributions

KG and ID participated in writing the manuscript, performing of the research, and data analysis. MV, MB, PK, and MM participated in data collection. MP participated in performing the research. All authors contributed to the article and approved the submitted version.
